# RISK OF PRENEOPLASTIC LESIONS IN MUCOSAL PROJECTIONS OF DIFFERENT SIZES OF THE COLUMNAR EPITHELIUM IN THE LOWER ESOPHAGUS

**DOI:** 10.1590/0102-672020220002e1674

**Published:** 2022-09-09

**Authors:** Hairton Copetti, Leonardo Copetti, Laura Copetti, Giulliano Danezi Felin, Giancarllo Danezi Felin, Carollina Danezi Felin, Fellipe Danezi Felin, Vitória Chiesa

**Affiliations:** 1 Universidade Federal de Santa Maria – Santa Maria (RS), Brazil; 2 Universidade Franciscana – Santa Maria (RS), Brazil; 3 Pontifícia Universidade Católica do Rio Grande do Sul – Porto Alegre (RS), Brazil; 4 Hospital Ernesto Dornelles – Porto Alegre (RS), Brazil; 5 Universidade Federal de Ciências da Saúde de Porto Alegre – Porto Alegre (RS), Brazil.

**Keywords:** Adenocarcinoma, Biopsy, Endoscopy, Barrett Esophagus, Esophageal Mucosa, Adenocarcinoma, Biópsia, Endoscopia, Esôfago de Barrett, Mucosa Esofágica

## Abstract

**BACKGROUND:**

Barrett's esophagus is an acquired condition that predisposes to the development of esophageal adenocarcinoma.

**AIMS:**

The aim of this study was to establish an association between the endoscopic and the histopathological findings regarding differently sized endoscopic columnar epithelial mucosa projections in the low esophagus, under 3.0 cm in the longitudinal extent.

**METHODS:**

This is a prospective study, including 1262 patients who were submitted to upper gastrointestinal endoscopy in the period from July 2015 to June 2017. The suspicious projections were measured and subdivided into three groups according to the sizes encountered (Group I: <0.99 cm; Group II: 1.0–1.99 cm; and Group III: 2.0–2.99 cm), and biopsies were then performed.

**RESULTS:**

There was a general prevalence of suspicious lesions of 6.42% and of confirmed Barrett's lesions of 1.17%, without a general significant statistical difference among groups. However, from Groups I and II to Group III, the differences were significant, showing that the greater the lesion, the higher the probability of Barrett's esophagus diagnosis. The absolute number of Barrett's lesions was 7, 9, and 6 for Groups I, II, and III, respectively.

**CONCLUSIONS:**

The findings led to the conclusion that even projections under 3.0 cm present a similar possibility of evolution to Barrett's esophagus. If, on the one hand, short segments are more prevalent, on the other hand, the long segments have the higher probability of Barrett's esophagus diagnosis, which is why biopsies are required in all suspicious segments.

## RESUMO

**RACIONAL:**

O esôfago de Barrett é uma condição adquirida que predispõe ao desenvolvimento de adenocarcinoma de esôfago.

**OBJETIVOS:**

Estabelecer uma associação entre os achados endoscópicos e histopatológicos em relação às projeções endoscópicas da mucosa epitelial colunar de diferentes tamanhos no esôfago, abaixo de 3,0 centímetros de extensão longitudinal.

**MÉTODOS:**

Foi realizado um estudo prospective incluindo 1262 pacientes submetidos à endoscopia digestiva alta, no período de julho de 2015 a junho de 2017. As projeções suspeitas foram medidas, subdivididas em 3 grupos de acordo com os tamanhos encontrados (Grupo I: <0,99 cm; Grupo II: 1,0 cm–1,99 cm; Grupo III: 2,0 cm–2,99 cm) e biópsias foram então realizadas.

**RESULTADOS:**

Houve prevalência geral de lesões suspeitas de 6,42% e de lesões de Barrett confirmadas de 1,17%, sem diferença estatística geral significativa entre os grupos. Porém, dos Grupos I e II, para o Grupo III, as diferenças foram significativas, mostrando que quanto maior a lesão, maior a probabilidade de diagnóstico de esôfago de Barrett. O número absoluto de lesões de Barrett foi 7, 9 e 6 para os grupos I, II e III, respectivamente.

**CONCLUSÕES:**

Os achados permitiram concluir que mesmo projeções abaixo de 3,0 cm apresentam possibilidade semelhante de evolução para o esôfago de Barrett. Se, por um lado os segmentos curtos são mais prevalentes, por outro os segmentos longos têm maior probabilidade de diagnóstico de esôfago de Barrett, razão pela qual são necessárias biópsias em todos os segmentos suspeitos.

## INTRODUCTION

Throughout the esophagus, the mucosa is lined by pavement, nonkeratinized, and stratified squamous epithelium, while the gastric mucosa is lined by columnar epithelium^9^.

**Figure 1:**
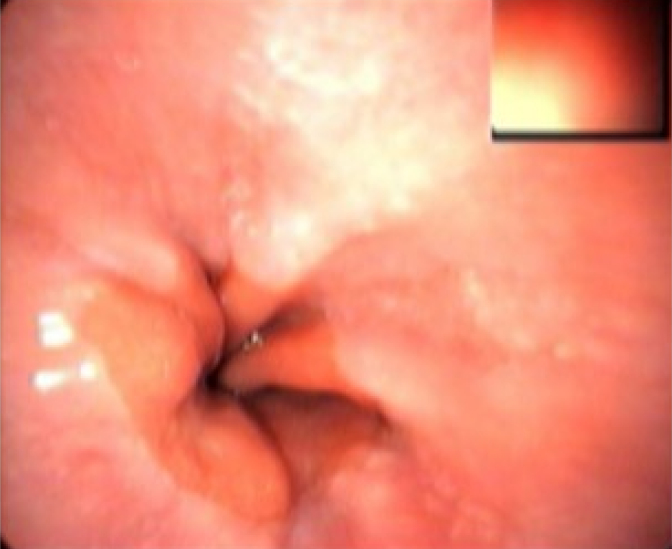
Esophagogastric junction.

The squamocolumnar junction is endoscopically observed as a transition from the pale pink epithelium in the esophagus to the red epithelium in the stomach (Figure 1). That abrupt transition line between epithelia is called Z-line and is of irregular shape. In normal conditions, the squamocolumnar junction and the esophagogastric junction are located at the same level^9^.

When the squamous epithelium in the esophagus is exposed to acid and/or bile reflux, a pathological situation present in gastroesophageal reflux disease, and the squamous cells in the esophageal mucosa are damaged by a chronic inflammatory process. The repair to that damage is, preferably, a replacement of squamous cells with metaplastic columnar cells, dislocating the squamocolumnar junction cranially^35^. This process of metaplastic cellular substitution is called Barrett's esophagus, which is a condition that predisposes to esophageal adenocarcinoma, as described later.

The precise sequence of events that leads to intestinal metaplasia has not been clearly established^11^. Similar to other premalignant conditions, Barrett's esophagus requires a genetic predisposition associated with environmental exposure. Metaplastic changes are most likely a protection mechanism in response to chronic inflammation or tissue lesion^18^.

Gastroesophageal reflux disease increases the probability of the development of Barrett's esophagus by 6–8 times^1^. Recent studies in the Western countries have shown that 20–25% of the adult population report symptoms related to reflux, at least once a week^20^.

It is hard to know the true prevalence of Barrett's esophagus because many individuals are asymptomatic and, thus, will never be evaluated. If the matter of prevalence already presents broad variations, the incidence is even harder to estimate. According to epidemiological studies at endoscopy units, when the examinations are performed without symptoms of reflux, Barrett's esophagus is observed in 0.05–2% of cases. However, when those symptoms are present, it is found in 5–15% of cases^29^.

**Figure 2:**
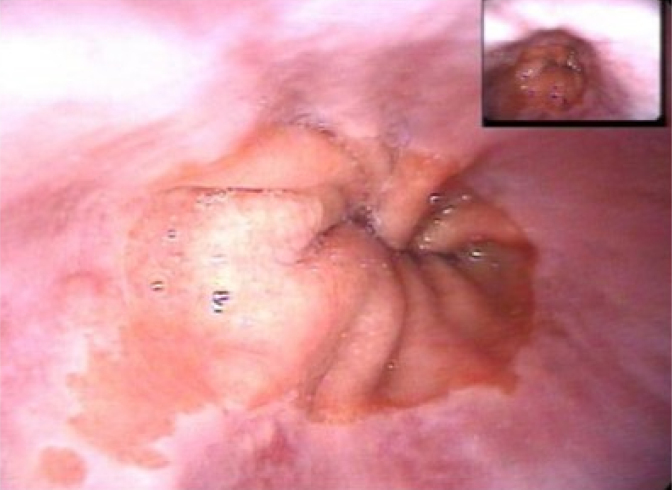
Mucosa projection of columnar epithelium.

The diagnosis of Barrett's esophagus is suspected when it is possible to observe, via upper gastrointestinal endoscopy, the presence of columnar mucosa in the esophagus in the shape of digit form projections, or segments that cover the circumference of the esophagus either partially or totally^12–33^ (Figure 2). Then, biopsies are performed in these areas, one in every quadrant, as well as in elevated or depressed areas, according to the Seattle protocol^40^. Finally, the diagnosis is confirmed when, in the histological analysis, columnar epithelium with specialized intestinal metaplasia and its characteristic Goblet cell is demonstrated^9–34^.

The most important aspect to be considered with Barrett's esophagus is the risk of potential development of esophageal adenocarcinoma^3,13^. It is estimated that the risk is 30–125 times higher than the general population^15^. The progression of Barrett's esophagus to adenocarcinoma depends on several factors, the most important one being histological grade. Nondysplastic Barrett's epithelium progresses at a rate of 3.86 per 1000 person-years, low-grade dysplasia progresses at a rate of 7.66 per 1000 person-years, and high-grade dysplasia progresses at 146 cases per 1000 person-years^40^.

If, on the one hand, adenocarcinoma is more frequent in longer Barrett's lesions, on the other hand, short and even ultrashort-segments are more prevalent^21^. Besides, it has been demonstrated that short segments, once developed, may expand in length^2^. Thus, it is clear that the extension of the metaplastic area has an effect on the risk of esophageal neoplasia, although its true degree of influence is not yet completely clear^19^.

Even though biopsies are widely recommended in any area with projections of columnar mucosa in the esophagus^36^, definitive and convincing studies correlating differently sized columnar epithelium and histopathological findings have not been found, especially in mucosa projections under 3.0 cm in longitudinal extent. Such studies might be able to indicate criteria for biopsies as to the size of these projections when diagnosing preneoplastic and/or neoplastic lesions. Therefore, the gravity of the evolution of Barrett's esophagus to esophageal adenocarcinoma, including short-segment, justifies research that correlates the size of suspicious mucosa projections under 3.0 cm, analyzed at 1 cm intervals, and histopathological findings, regardless of the clinical indication for endoscopic examination.

## METHODS

### Type of study

It consisted of a prospective, experimental study, of diagnostic investigation, by a series of consecutive cases in three different locations — a private physician's office and two public hospitals in Santa Maria, RS, Brazil.

### Groups of patients

From July 2015 to June 2017, 1,262 upper gastrointestinal endoscopy exams were performed. Those with suspicious areas of columnar mucosa in the low esophagus were classified into three groups:

- Group 1 – projections up to 0.99 cm;
- Group 2 – 1.00–1.99 cm; and
- Group 3 – 2.0–2.99 cm.

### Exam protocol

The Informed Consent Form was approved by the Ethics Committee of Universidade Federal de Santa Maria, Santa Maria, RS, Brazil (number: 1.088.491) and Plataforma, Brazil. It was presented to patients 24 h before the examination and additional questions were duly answered. The Confidentiality Agreement was signed by the author.

The examinations were performed in three different locations, by the same endoscopist who utilized video endoscopes PENTAX model EG-2931K and OLYMPUS Exera II. Images were captured using the Laudo & Imagem 3.1 capture system, developed by AKTTOM Systems. Preparation of patients consisted of an 8-h fast period, simethicone drops orally; immediately before the exam, xylocaine spray in the oropharynx and intravenous midazolam with dosage adjusted to weight, age, and sedation level for each patient — in general, dosage ranged between 2 and 5 mg IV.

The examinations were performed employing the habitual technique for upper gastrointestinal endoscopies.

**Figure 3:**
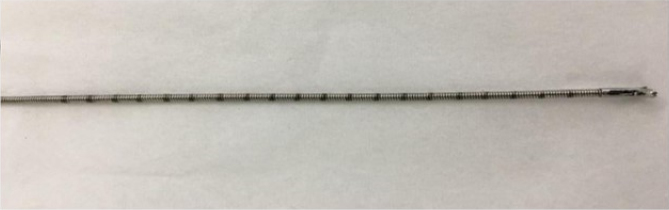
Measuring clamp.

**Figure 4:**
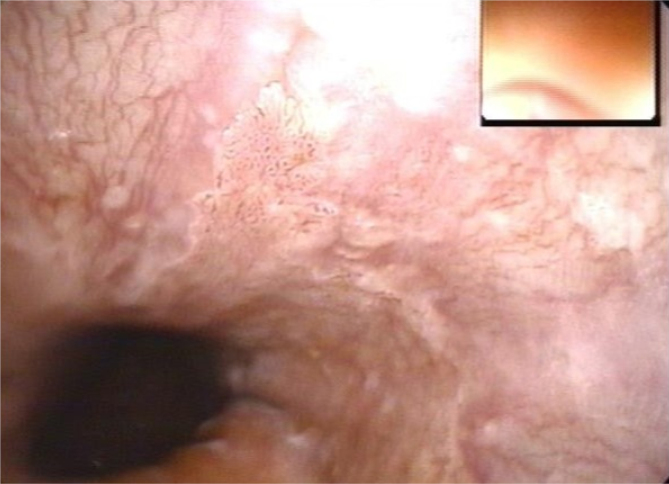
Acetic acid coloring of small area.

**Figure 5:**
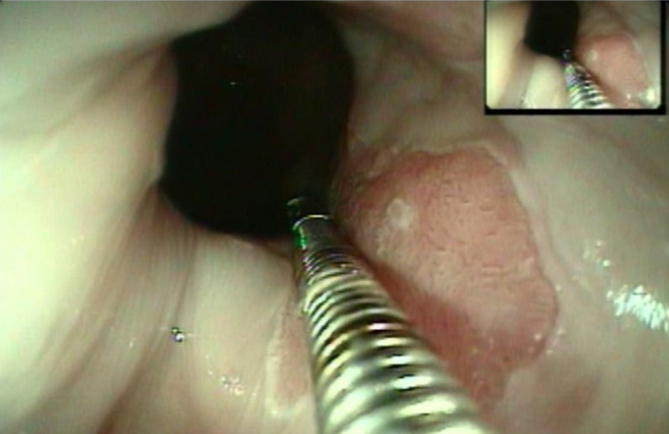
Measurement with graded clamp.

**Figure 6:**
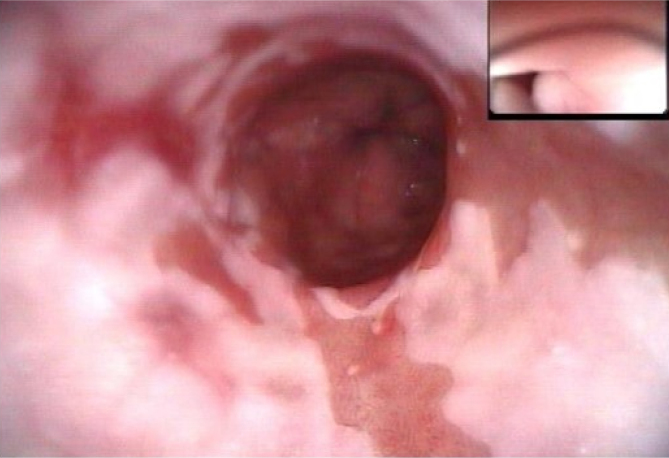
Area suspicious of Barrett's.

**Figure 7:**
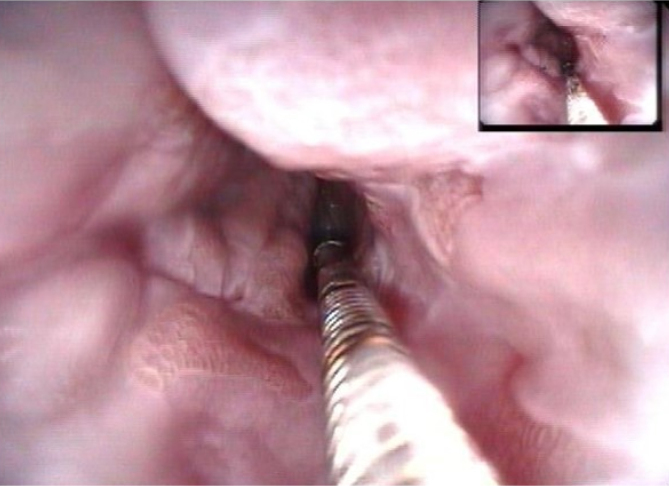
Measurement after acetic acid.

Suspicious areas were identified by mucosa projection, from the top of the gastric folds to the columnar mucosa, more reddish, and vascularized in the low esophagus. At this moment, insufflation was diminished for better identification of the gastric folds^29^. Then, a 1.5% application of acetic acid was used for coloring^16^. Once the suspicious area was identified, a previously laser-graded biopsy clamp with 0.5 cm intervals, designed by the author, was introduced through the gastroscope working canal; the projection was measured and then biopsies were performed following the Seattle Protocol^41^ (Figures 3–7).

**Figure 8:**
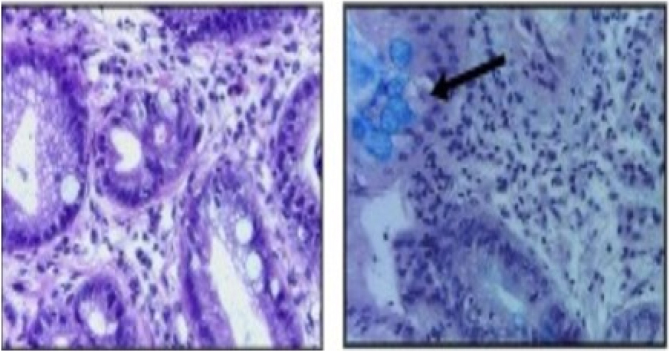
Goblet cells after Alcian blue staining.

The fragments were preserved in formalin and sent to the pathologist for analysis, following habitual techniques for preparation and staining, hematoxylin-eosin, and Alcian blue (Figure 8).

### Inclusion criteria

All consecutive patients coming in with a request for upper gastrointestinal endoscopy were included, regardless of the clinical indication.

### Exclusion criteria

Patients who declined to participate, esophageal obstruction, post-esophagectomy and bariatric surgery patients, projections of 3 cm, uncooperative patients during examination, and patients aged under 18 years were excluded from the study.

## RESULTS

The number of suspected lesions during endoscopic examination was 81, where 59 subjects (72.8%) did not confirm for Barrett's esophagus after histological examination, and 22 (27.2%) were confirmed, representing 1.74% of the sample total (Table 1).

**Table 1:** Number of suspected cases in relation to total number of patients submitted to upper gastrointestinal endoscopy.

Patients	Total	%
Submitted to UGE	1,262	100.0
Suspicious lesions	81	6.42
Group I	31	2.46
Group II	39	3.09
Group III	11	0.87

UGE: Upper Gastrointestinal Endoscopy.

The chi-square test was used to analyze the association between confirmed cases and the differently sized lesions; it is concluded that among the three groups, there is no association between the confirmations by biopsy and the size of lesions (p=0.089). However, between Groups I and III and II and III, if Groups I and II are merged and compared to Group III, there is a significant increase in the proportion of confirmed cases (p=0.049, 0.044, and 0.028, respectively). In the 22 confirmed cases of Barrett's esophagus, the highest prevalence within groups was 54.5% in Group III (Table 2).

**Table 2:** Results of lesions per group – confirmed cases in relation to the suspected cases.

Lesions	Group I	Group II	Group III	Total
Suspected	31	39	11	81
Confirmed	7	9	6	22
Percentage in relation to groups	22.6	23.1	54.5	27.2

In relation to the number of biopsies, a statistically significant difference was observed among the groups (p<0.001). However, when comparing the number of biopsies between confirmed and unconfirmed cases, there is no statistical difference (p=0.303) (Tables 3 and 4).

**Table 3:** Mean number of biopsies and standard deviation per group (p<0001).

	Mean biopsies	SD
Group I	2.26	0.89
Group II	3.18	1.00
Group III	4.27	1.10
Total	2.98	1.17

SD: standard deviation.

**Table 4:** Mean number of biopsies and standard deviation of confirmed and unconfirmed cases (p=0.145).

	Mean biopsies	SD
Unconfirmed	2.86	1.04
Confirmed	3.27	1.45

SD: standard deviation.

## DISCUSSION

Barrett's esophagus is the greatest known link so far to esophageal carcinoma, as widely demonstrated in the literature. Despite the numerous studies and publications in the scientific community, it remains controversial and with questions to be clarified, including diagnostic suspicion, prevalence, incidence, diagnostic criteria, research methods, result interpretation, and treatment prevalence of suspicious lesions which will or will not be later confirmed as Barrett's esophagus.

In this analysis, among the 1,262 subjects examined, there was endoscopic suspicion in 81 cases, which makes up 6.42% of the sample. A 13-year-long population study in Northern Ireland with 261,725 endoscopies found suspected Barrett's in 9.5%, considering mucosa projections of all sizes^4^.

Only longitudinal mucosa projections were measured. Although somehow often cited in the literature, the Prague C & M criteria^30^ and clinically relevant, this measurement system may fail in identifying short-segment Barrett's esophagus^19^. Endoscopic tubes are marked every 5.0 cm and measurements are taken using the superior dental arch as a reference. In an attempt to use a less rudimentary measurement system with more accuracy, the author of this thesis projected a biopsy clamp, one that could be sterilized and laser-graded at 0.5 cm intervals. It is possible that this measurement system marks an advance in endoscopic grading.

After histopatological analysis of the 81 cases, only 22 (or 27.2%) were confirmed as Barrett's, which corresponded to a general percentage of 1.74% of the sample, well below the confirmation percentage of 49% of suspicious projections and 4.7% of the total sample referred to by Coleman et al.^20^. The search for suspicious lesions is possibly influenced by the examiner's level of training and interest. Coleman et al.^4^ considered that the presence of metaplastic transformation is not a condition required for the diagnosis of Barrett's esophagus.

Considering that the three groups evaluated in this research could be historically classified within the large group of short Barrett's, the percentage found shows a tendency of higher confirmation in larger lesions. As observed, Group III had a higher confirmation percentage at 54.5%. Similarly, these findings were recorded in Richter's publication, where 75% of long projections and 30% of short projections were confirmed^22^.

In relation to the confirmed Barrett's esophagus, the results point to the conclusion that there is no significant difference among Groups I–III. However, between Groups I and III and II and III, there is a significant increase in the proportion of confirmed cases, and, if Groups I and II – which correspond to smaller lesions — are merged, the percentage for those segments is around 1.26%, well over the 0.48% percentage of Group III. In a series of 1,000 random endoscopies, in Swedish adults, 1.6% of patients presented with Barrett's esophagus, 0.5% with long segments and 1.1% with short segments, 2.3% in patients with symptoms of reflux, and 1.4% for asymptomatic individuals^25^.

Patients with symptoms of reflux disease seem to have a higher prevalence of Barrett's esophagus^5^, with one study identifying 13.2% among symptomatic individuals^41^. Other studies confirm that information estimating it to be 1–2% in all patients submitted to endoscopy for any indication and 5–15% in all patients that present symptoms of gastroesophageal reflux disease^29,37,42^. The prevalence of short segments in some published series ranged from 2 to 12% in patients submitted to upper digestive endoscopies with biopsies^31^. The prevalence of long segments (3 cm) was 5%, while short segments (3 cm) fluctuated from 6 to 12%, in a variety of studies^8^.

One large population study in Santa Maria University Hospital, located in the south of Brazil, involving approximately 5,000 patients, has set the total prevalence of Barrett's esophagus at 1.7% (5.6% in patients with gastroesophageal reflux disease)^7^.

As demonstrated in previously mentioned references, the 1.74% prevalence found by the author of this study is similar to the results reported by most publications, especially in those among the general population, without considering risk groups.

It is important to note that, to the exception of a few studies, there is no stratification by groups of different sizes, which makes an accurate comparison to the data in the literature incomplete. Besides the fact that the author of this study took into consideration the general population, regardless of the clinical indication for endoscopy, another factor that certainly contributed to some lower prevalence percentages reported in the literature was the criteria for histological diagnosis.

The diagnostic criteria for Barrett's esophagus, in this study, were considered the substitution of squamous for columnar epithelium with intestinal metaplasia and the characteristic Goblet cell, such as advocated by the Brazilian Endoscopy Society and the American College of Gastroenterology. These positions are not shared by other societies, including the British Society of Gastroenterology, which does not require the presence of intestinal metaplasia for confirmation^34^.

The key point in recommending the requirement of metaplasia is the high incidence of esophageal adenocarcinoma among patients with intestinal metaplasia when compared to patients who have a diagnosis of Barrett's esophagus without metaplasia^17^. Evidently, the criteria of diagnostic requirement for the presence of Goblet cells decrease the prevalence of Barrett's esophagus in comparison to not requiring it, a situation that considers the presence of any layer of columnar mucosa^24^. If any columnar epithelium is considered Barrett's esophagus, it is possible that 100% of long segments and 95% of short segments are also classified as Barrett^16^.

A comparative study to determine the optimal number of biopsies for diagnosis verified that when biopsies were increased from 4 to 8 and 16 or more, the diagnosis of Barrett's metaplasia increased from 34.7 to 67.9% and to 100%, respectively^14^. Whenever possible, the author of this thesis followed the Seattle protocol, which refers to obtaining random biopsies in the four quadrants, beyond elevated or irregular areas^41^. However, studies demonstrate that even when properly following the protocol, samples could be obtained from only 4 to 6% of the Barrett's esophagus area^14^.

Out of 22 patients confirmed for Barrett's esophagus, no cases of dysplasia and/or adenocarcinoma were diagnosed. This is new and a contribution to the bibliography. In a meta-analysis involving 11,387 patients with Barrett's esophagus, the prevalence of low-grade dysplasia was around 13%^39^.

A retrospective cohort study of the natural history of low-grade dysplasia demonstrated that 85% of patients with an initial diagnosis of low-grade dysplasia could actually be downstaged to no dysplasia or indefinite for dysplasia after an expert pathologist review^6^. For these reasons, some medical societies recommend that dysplasia be confirmed by a second specialist before therapies are initiated^10^.

Shields et al. demonstrated that a great number of studies do not support a linear relationship between Barrett's extension and risk of cancer^32^. A large meta-analysis and systematic review showed that the mean incidence of cancer in Barrett's esophagus was 6.1 per 100,000 person-years, 6.7 in longer segments, and 6.1 in shorter segments. Although other studies indicate that patients with longer segments have a higher risk of developing cancer, that review did not demonstrate differences in general incidence among patients with long or short segments^27,43^.

Histological confirmation of dysplasia remains the only acceptable factor in predicting progression to cancer^38^ and is defined by the presence of neoplastic epithelium, with cellular atypia, and with no invasion of the basement membrane^23^.

Barrett's esophagus is the known precursor of a large majority of adenocarcinomas^28^. There is substantial evidence that the extension of the Barrett's segment is an important risk factor; however, that is not a consensus. In some articles and meta-analyses, the progression risk in short segments has been similar to that in long segments^26^.

## CONCLUSIONS

Considering Barrett's esophagus a preneoplastic lesion, the findings of this research allowed us to conclude that the projections of columnar epithelium in the low esophagus (2.99 cm), evaluated at 1 cm intervals, present the same risk of having preneoplastic or neoplastic lesions among themselves. If, on the one hand, shorter projections are more frequent, on the other hand, longer ones present a higher percentage of confirmation, resulting in a very similar absolute numbers of cases of Barrett's esophagus. Thus, biopsies are widely recommended and must be systematically performed in any area with projections of columnar mucosa, despite the projection size.
